# Development, testing and use of data extraction forms in systematic reviews: a review of methodological guidance

**DOI:** 10.1186/s12874-020-01143-3

**Published:** 2020-10-19

**Authors:** Roland Brian Büchter, Alina Weise, Dawid Pieper

**Affiliations:** Institute for Research in Operative Medicine (IFOM), Faculty of Health - School of Medicine, Witten/Herdecke University, Ostmerheimer Str. 200, 51109 Cologne, Germany

**Keywords:** Systematic review methods, Evidence synthesis, Data extraction

## Abstract

**Background:**

Data extraction forms link systematic reviews with primary research and provide the foundation for appraising, analysing, summarising and interpreting a body of evidence. This makes their development, pilot testing and use a crucial part of the systematic reviews process. Several studies have shown that data extraction errors are frequent in systematic reviews, especially regarding outcome data.

**Methods:**

We reviewed guidance on the development and pilot testing of data extraction forms and the data extraction process. We reviewed four types of sources: 1) methodological handbooks of systematic review organisations (SRO); 2) textbooks on conducting systematic reviews; 3) method documents from health technology assessment (HTA) agencies and 4) journal articles. HTA documents were retrieved in February 2019 and database searches conducted in December 2019. One author extracted the recommendations and a second author checked them for accuracy. Results are presented descriptively.

**Results:**

Our analysis includes recommendations from 25 documents: 4 SRO handbooks, 11 textbooks, 5 HTA method documents and 5 journal articles. Across these sources the most common recommendations on form development are to use customized or adapted standardised extraction forms (14/25); provide detailed instructions on their use (10/25); ensure clear and consistent coding and response options (9/25); plan in advance which data are needed (9/25); obtain additional data if required (8/25); and link multiple reports of the same study (8/25). The most frequent recommendations on piloting extractions forms are that forms should be piloted on a sample of studies (18/25); and that data extractors should be trained in the use of the forms (7/25). The most frequent recommendations on data extraction are that extraction should be conducted by at least two people (17/25); that independent parallel extraction should be used (11/25); and that procedures to resolve disagreements between data extractors should be in place (14/25).

**Conclusions:**

Overall, our results suggest a lack of comprehensiveness of recommendations. This may be particularly problematic for less experienced reviewers. Limitations of our method are the scoping nature of the review and that we did not analyse internal documents of health technology agencies.

## Background

Evidence-based medicine has been defined as the integration of the best-available evidence and individual clinical expertise [1]. Its practice rests on three fundamental principles: 1) that knowledge of the evidence should ideally come from systematic reviews, 2) that the trustworthiness of the evidence should be taken into account and 3) that the evidence does not speak for itself and appropriate decision making requires trade-offs and consideration of context [2]. While the first principle directly speaks to the importance of systematic reviews, the second and third have important implications for their conduct. The second principle implies that systematic reviews should be based on rigorous, bias-reducing methods. The third principle implies that decision makers require sufficient information on the primary evidence to make sense of a review’s findings and apply them to their context.

Broadly speaking, a systematic review consists of five steps: 1) formulating a clear question, 2) searching for studies able to answer this question, 3) assessing and extracting data from the studies, 4) synthesizing the data and 5) interpreting the findings [3]. At a minimum, steps two to five rely on appropriate and thorough data collection methods. In order to collate data from primary studies, standardised data collection forms are used [4]. These link systematic reviews with primary research and provide the foundation for appraising, analysing, summarising and interpreting a body of evidence. This makes their development, pilot testing and application a crucial part of the systematic reviews process.

Studies on the prevalence and impact of data extraction errors have recently been summarised by Mathes and colleagues [5]. They identified four studies that looked at the frequency of data extraction errors in systematic reviews. The error rate for outcome data ranged from 8 to 63%. The impact of the errors on summary results and review conclusions varied. In one of the studies the effect size from the meta-analytic point estimates changed by more than 0.1 in 70% of cases (measured as standardised differences in means) [6]. Considering that most interventions have small to moderate effects, this can have a large impact on conclusions and decisions. Little research has been conducted on extraction errors relating to non-outcome data.

The importance of a rigorous data extraction process is not restricted to outcome data. As previously mentioned, users of systematic reviews need sufficient information on non-outcome data to make sense of the underlying primary studies and assess their applicability. Despite this, many systematic reviews do not sufficiently report this information. In one study almost 90% of systematic reviews of interventions did not provide the information required for treatments to be replicated in practice – compared to 35% of clinical trials [7]. While there are several possible reasons for this – including the quality of reporting – insufficient data collection forms or procedures may to contribute to the problem.

Against this background, we sought to review the guidance that is available to systematic reviewers for the development and pilot testing of data extraction forms and the data extraction process, these being central elements in systematic reviews.

## Methods

This project was conducted as part of a dissertation, for which an exposé is available in German. We did not publish a protocol for this descriptive analysis, however. As there are no specific reporting guidelines for this type of methodological review, we reported our methods in accordance with the PRISMA statement as applicable [8].

Systematic reviews are conducted in a variety of different contexts – most notably as part of dissertations or academic research projects, as standalone projects, by health technology assessment (HTA) agencies and by systematic review organisations (SROs). Thus, we looked at a broad group of sources to identify recommendations:

1. Methodological handbooks from major SROs
2. Textbooks aimed at students and researchers endeavouring to conduct a systematic review
3. Method documents from HTA agencies
4. Published journal articles making recommendations on how to conduct a systematic review or how to develop data extraction forms

While the sources that we searched mainly focus on medicine and health, we did not exclude other health-related areas such as the social sciences or psychology.

### Data sources

Regarding the methodological handbooks from SROs, we considered the following to be the most relevant to our analysis:

- The Centre for Reviews and Dissemination’s guidance for undertaking reviews in health care (CRD guidance)
- The Cochrane Handbook of Systematic Reviews of Interventions (Cochrane Handbook)
- The Institute of Medicine’s Finding What Works in Health Care: Standards for Systematic Reviews (IoM Standards)
- The Joanna Briggs Institute’s Reviewer Manual (JBI Manual)

The list of textbooks was based on a recently published article that reviewed systematic review definitions used in textbooks and other sources [9]. The authors did not carry out a systematic search for textbooks, but included textbooks from a broad range of disciplines including medicine, nursing, education, health library specialties and the social sciences published between 1998 and 2017. These textbooks included information on data extraction in systematic reviews, but none of them focussed on this topic exclusively.

Regarding the HTA agencies, we compiled a list of all member organisations of the European Network for Health Technology Assessment (EUnetHTA), the International Network of Agencies for Health Technology Assessment (INAHTA), Health Technology Assessment international (HTAi) and the Health Technology Assessment Network of the Americas (Red de Evaluación de Tecnologías en Salud de las Américas – RedETSA). The reference month for the compilation of this list was January 2019, the list is included in additional file 1. We searched these websites for potentially relevant documents and downloaded these. We then reviewed the full texts of all documents for eligibility and included those that fulfilled our inclusion criteria. The website searches and the full text screening of the documents were conducted by two authors independently (RBB and AW). Disagreements were resolved by discussion. We also planned to include the newly founded Asia-Pacific HTA network (HTAsiaLink), but the webpage had not yet been launched during our research period.

To identify relevant journal articles, we first searched the Scientific Resource Center’s Methods Library (SRCML). This is a bibliography of publications relevant to evidence synthesis methods which was maintained until the third quarter of 2017 and has been archived as a RefWorks library. Because the SRCML is no longer updated, we conducted a supplementary search of Medline from the 1st of October 2017 to the 12th of December 2019. Finally, we searched the Cochrane Methodology Register (CMR), a reference database of publications relevant to the conduct of systematic reviews that was curated by the Cochrane Methods Group. The CMR was discontinued on the 31st of May 2012 and has been archived. Due to the limited search and export functions of these archived SRCML and CMR, we used pragmatic search methods for these sources. The search terms that were used for the databases searches are included in additional file 2. The titles and abstracts from the database searches and the full texts of potentially relevant articles were screened for eligibility by two authors independently (RBB and AW). Disagreements were resolved by discussion or, if this was unsuccessful, arbitration with DP.

### Inclusion criteria

To be eligible for inclusion in our review, documents had to fulfil the following criteria:

- Published method document (e.g. handbook, guidance, standard operating procedure, manual), academic textbook or journal article
- Include recommendations on the development or piloting of data extraction forms or the data extraction process in systematic reviews
- Available in English or German

We excluded empirical research on different data extraction methods as well as papers on technical aspects, because these have been reviewed elsewhere [10–12]. This includes, for example, publications on the merits and downsides of different types of software (word processors, spreadsheets, database or specialised software) or the use of pencil and paper versus electronic extraction forms. We also excluded conference abstracts and other documents not published in full.

For journal articles we specified the inclusion and exclusion criteria more narrowly as this group includes a much broader variety of sources (for example we excluded “primers”, i.e. articles that provide an introduction to reading or appraising a systematic review for practitioners). The full list of inclusion and exclusion criteria for journal articles is published in additional file 2.

### Items of interest

We looked at a variety of items relevant to three categories of interest:

1. the development of data extraction forms,
2. the piloting of data extraction forms and
3. the data extraction process.

To our knowledge, no comprehensive list of potentially relevant items exists. We therefore developed a list of potentially relevant items based on iterative reading of the most influential method handbooks from SROs (see above) and our personal experience. The full list of items included in our extraction form is reported in additional file 3 together with a proposed rationale for each item.

We did not examine recommendations regarding the specific information that should be extracted from studies, because this depends on a review’s question. For example, reviewers might choose to include information on surrogate outcomes in order to aid interpretation of effects or they might choose not to, because they often poorly correlate with clinical endpoints and the researchers are interested in patient-relevant outcomes [13, 14]. Furthermore, the specific information that is extracted for a review depends on the area of interest with special requirements for complex intervention or adverse effects reviews, for example [15]. For the same reason, we did not examine recommendations regarding specific methodological or statistical aspects. For instance, when a generic inverse variance meta-analysis is conducted, standard errors are of interest, whereas in other cases standard deviations may be preferably extracted.

### Data extraction

One author developed the first draft of the data extraction form to gather information on the items of interest. This was reviewed by DP and complemented and revised after discussion. We collected bibliographic data, direct quotations on recommendations from the source text and page numbers.

Each item was coded using a coding scheme of five possible attributes:

- recommendation for the use of this method
- recommendation against the use of this method
- optional use of this method
- a general statement on this method without a recommendation
- method not mentioned

For some items descriptive information was of additional interest. This included specific recommendations on the sample of studies that should be used to pilot the data extraction form or the experience or expertise of the reviewers that should be involved. Descriptive information was copied and pasted into the form. The form also included an open field for comments in case any additional items of interest were identified.

One author (RBB) extracted the information of interest from the included documents using the final version of the extraction form. A second author double-checked the information for each of the extracted items (AW). Discrepancies were resolved by discussion or by arbitration with DP.

During extraction, one major change was required to the form. Initially, we considered quantifying agreement only during the piloting phase of an extraction form, but later realised that some sources recommended this for the extraction phase of a review. We thus added items on quantifying agreement to this category.

### Data analysis

We separately analysed and reported the four groups of documents (handbooks from SROs, documents from HTA agencies, textbooks and journal articles) and the three categories of interest (development, piloting and extraction). We summarised the results of our findings descriptively. We also aggregated the results across sources for each item using frequencies. Additional information is presented descriptively in the text.

In our primary analysis we only included documents that made recommendations for interventional reviews or generic recommendations. We did this because almost all included documents focussed on these types of reviews and, more importantly, to avoid inclusion of multiple recommendations from one institution. This was particularly relevant for the Joanna Briggs Institute’s Reviewer Manual which at the time of our analysis had 10 separate chapters on a variety of different systematic review types. The decision to restrict the primary analysis to documents focussing on interventional reviews and generic documents was made post hoc. Results for other types of reviews (e.g. scoping reviews, umbrella reviews, economic reviews) are presented as a secondary analysis.

## Results

We identified and searched 158 webpages of HTA agencies via the member lists of EUnetHTA, INAHTA, HTAi and RedETSA (see additional file 1). This resulted in 155 potentially relevant method documents from 67 agencies. After full text screening, 6 documents remained that fulfilled our inclusion criteria. The database searches resulted in 2982 records. After title and abstract screening, 15 potentially relevant full texts remained. Of these 5 fulfilled our inclusion criteria. A PRISMA flow chart depicting the screening process for the database searches is provided in additional file 2 and for the HTA method documents in additional file 1.

In total, we collected data from 14 chapters in 4 handbooks of SROs [16–19], 11 textbooks [3, 20–29], 6 method documents from HTA agencies [30–35] and 5 journal articles [36–40]. Additional file 4 lists all documents that fulfilled our inclusion criteria. In our primary analysis we describe recommendations from a total of 25 sources: 4 chapters from 4 SRO handbooks, 11 textbooks, 5 method documents from HTA agencies and 5 journal articles. Our secondary analysis on recommendations for non-interventional systematic reviews is included in Additional file 5 and the detailed results for the primary analysis in Additional file 6.

### Synthesis of the primary analysis

In sum, we analysed recommendations from 25 sources in our primary analysis. The most frequent recommendations on the development of extraction forms are to use customised or adapted standardised extraction forms (14/25); provide detailed instructions on their use (10/25); ensure clear and consistent coding and response options (9/25); plan in advance which data are needed (9/25); obtain additional data if required (8/25); and link multiple reports of the same study (8/25).

The most frequent recommendations on piloting extractions forms are that forms should be piloted on a sample of studies (18/25); and that data extractors should be trained in the use of the forms (7/25).

The most frequent recommendations on data extraction are that data extraction should be conducted by at least two people (17/25); that independent parallel extraction should be used (11/25); and that procedures to resolve disagreements between data extractors should be in place (14/25).

To provide a more comprehensible overview and illustrate areas where guidance is sparse, we have aggregated the results for definite recommendations (excluding optional recommendations or general statements) in Tables 1, 2 and 3. To avoid any misconceptions, we emphasise that by aggregating these results we by no means suggest that all items are of equal importance. Some are in fact mutually exclusive or interconnected.

**Table 1:** Recommendations for the development of data collection forms

Source group	Item									
	Plan in advance which data are needed	Develop a customized or adapted form	Use generic form	Ensure consistent and clear coding and response options	Provide detailed instructions	Involve reviewers with complementary expertise	Involve reviewers with experience in systematic review methods	Link multiple reports of the same study	Develop mechanism for recording, assessing and correcting data entry errors	Develop a strategy for obtaining data
**Handbooks from SROs**	3/4	3/4	1/4	2/4	2/4	1/4	1/4	4/4	1/4	1/4
**Textbooks on conducting systematic literature reviews**	2/11	6/11	0/11	4/11	3/11	1/11	1/11	3/11	0/11	4/11
**Guidance documents from HTA agencies**	1/5	2/5	0/5	1/5	3/5	0/5	0/5	1/5	0/5	2/5
**Recommendations published in journal articles**	3/5	3/5	0/5	2/5	2/5	1/5	1/5	0/5	2/5	1/5
**Total**	9/25	14/25	1/25	9/25	10/25	3/25	3/25	8/25	3/25	8/25

**Table 2:** Recommendations regarding the piloting of data collection forms

Source group	Item									
	Train data extractors in using the form	Pilot test form using a sample of studies	(Partially) repeat piloting if major changes are made during the review	In case of modifications to the data collection form, re-check reports that have already undergone data extraction	Involve reviewers with complementary expertise	Involve reviewer with experience in systematic review methods	Quantify agreement using a reliability measure such as Cohen’s kappa	If agreement is quantified, do this only for critical items	Repeat the piloting process until a specified agreement is reached	Informally consider reliability of coding while piloting
**Handbooks from SROs**	1/4	3/4	1/4	1/4	0/4	0/4	0/4	0/4	0/4	0/4
**Textbooks on conducting systematic literature reviews**	3/11	8/11	0/11	0/11	0/11	0/11	0/11	0/11	0/11	0/11
**Guidance documents from HTA agencies**	0/5	3/5	0/5	0/5	0/5	0/5	0/5	0/5	0/5	0/5
**Recommendations published in journal articles**	3/5	4/5	0/5	0/5	0/5	0/5	0/5	0/5	0/5	1/5
**Total**	7/25	18/25	1/25	1/25	0/25	0/25	0/25	0/25	0/25	1/25

**Table 3:** Recommendations regarding data extraction

Source group	Item												
	Data extraction should be conducted by at least two people	Data extraction by at least two people independently (parallel extraction)	Data extracted by one individual accuracy checks by a second (double-checking)	Parallel extraction for critical items and double-checking for non-critical items	Validation of a random sample of data by a third investigator	Data extraction by reviewers with complementary expertise	Data extraction by at least one reviewer with expertise in systematic review methods	Quantify agreement using a reliability measure such as Cohen’s kappa	If agreement is quantified, do this only for critical items	Informally consider reliability of coding throughout the review process	Explicit procedures or rules for resolving disagreements	Report who was involved in data extraction	Document disagreements and how they were resolved
**Handbooks from SROs**	4/4	3/4	0/4	0/4	0/4	1/4	0/4	0/4	1/4	2/4	4/4	2/4	2/4
**Textbooks on conducting systematic literature reviews**	6/11	4/11	0/11	0/11	0/11	1/11	1/11	1/11	0/11	0/11	5/11	0/11	1/11
**Guidance documents from HTA agencies**	4/5	1/5	1/5	0/5	0/5	0/5	0/5	0/5	0/5	0/5	3/5	1/5	0/5
**Recommendations published in journal articles**	3/5	3/5	0/5	0/5	1/5	0/5	0/5	0/5	0/5	1/5	2/5	0/5	1/5
**Total**	17/25	11/25	1/25	0/25	1/25	2/25	1/25	1/25	1/25	3/25	14/25	3/25	4/25

The following sections provide details for each groups of documents sorted by the three categories of interest.

### Handbooks of systematic review organisations

#### Category: development of extraction forms

Three handbooks recommend that reviewers should plan in advance which data to extract [16–18]. Furthermore, three recommended that reviewers develop a customized data extraction form or adapt an existing form to meet the specific review needs [17–19]. In contrast, the JBI recommends use of their own standardised data extraction form, but allows reviewers to use others, if this is justified and the forms are described [16]. All four handbooks recommend that reviewers link multiple reports of the same study to avoid multiple inclusions of the same data [16–19]. Three handbooks make statements on strategies for obtaining unpublished data [16–18]. The Cochrane Handbook recommends contacting authors to obtain additional data, while the CRD guidance makes a general statement in light of the chances of success and resources available. The JBI manual makes this optional but requires the systematic reviewers to report whether authors of included studies are contacted in the review protocol.

Two handbooks recommend that the data collection form includes consistent and clear coding instructions and response options and that data extractors are provided with detailed instructions on how to complete the form [17, 18]. The Cochrane Handbook also recommends that the entire review team should be involved in the development of the data extraction form and that this should include authors with expertise in the content area, review methods, statisticians and data extractors. The Cochrane Handbook also recommends that reviewers check compatibility of electronic forms or data systems with analytical software and ensure methods are in place to record, assess and correct data entry errors.

#### Category: piloting of extraction forms

Three handbooks recommended that authors pilot test their data extraction form [17–19]. The Cochrane Handbook recommends that “several people” are involved and “at least a few articles” used. The CRD guidance states that “a sample of included studies” should be used for piloting. The Cochrane Handbook also recommends that data extractors are trained; that piloting may need to be repeated if major changes to the extraction form are made during the review process; and that reports that have already been extracted should be re-checked in this case. None of the handbooks makes an explicit recommendation on who should be involved in piloting the data extraction form or their expertise. Furthermore, none of the handbooks makes a recommendation on quantifying agreement during the piloting process or using a quantified reliability threshold that should be reached before beginning the extraction process.

#### Category: data extraction

All handbooks recommend that data should be extracted by at least two reviewers (dual data extraction) [16–19]. Three handbooks recommend that data are extracted by two reviewers independently (parallel extraction) [16, 18, 19], one also considers it acceptable that one reviewer extracts the data and a second reviewer checks it for accuracy and completeness (double-checking) [17]. Furthermore, two of the handbooks make an optional recommendation that independent parallel extraction could be done only for critical data such as risk of bias and outcome data, while non-critical data is extracted by a single reviewer and double-checked by a second reviewer [18, 19]. The Cochrane Handbook also recommends that data extractors have a basic understanding of the review topic and knowledge of study design, data analysis and statistics [18].

All handbooks recommend that reviewers should have procedures in place to resolve disagreements arising from dual data extraction [16–19]. In all cases discussion between extractors or arbitration with a third person are suggested. The Cochrane Handbook recommends hierarchical use of these strategies, while the other sources do not specify this [18]. Of note, the IoM Standards highlights the need for a fair procedure that ensures both reviewers judgements are considered in case of a power or experience asymmetry [19]. The Cochrane Handbook also recommends that disagreements that remain unresolved after discussion, arbitration or contact with study authors should be reported in the systematic review [18].

Two handbooks recommend to informally consider the reliability of coding throughout the review process [17, 18]. These handbooks also mention the possibility of quantifying agreement of the extracted data. The Cochrane Handbook considers this optional and recommends it only for critical outcomes such as risk of bias assessments or key outcome data, if done [18]. The CRD guidance mentions this possibility without making a recommendation [17]. Two handbooks recommend that reviewers document disagreements and how they were resolved [17, 18] and two recommend reporting who was involved in data extraction [18, 19]. The IoM Standards specify this in that the number of individual data extractors and their qualifications should be reported in the methods section of the review [19].

### Textbooks on conducting systematic reviews

#### Category: development of extraction forms

Regarding the development of data extraction forms, the most frequent recommendation in the analysed textbooks is that reviewers should develop a customized extraction form or adapt an existing one to suit the needs of their review (6/11) [20, 21, 23, 24, 26, 29]. Two textbooks consider the choice between customized and generic or pre-existing extraction forms optional [3, 25].

Many of the textbooks also make statements on unpublished data (7/11). Most of them recommend that reviewers develop a strategy for obtaining unpublished data (4/11) [24–26, 29]. One textbook makes an optional recommendation on obtaining unpublished data and mentions the alternative of conducting sensitivity analysis to account for missing data [3]. Two textbooks make general statements regarding missing data without a compulsory or optional recommendation [22, 23].

Four textbooks recommend that reviewers ensure consistent and easy coding rules and response options in their data collection form [3, 22, 25, 29]; three to provide detailed instruction on how to complete the data collection form [22, 24, 25]; and three to link multiple reports of the same study [3, 24, 26]. One textbook discusses the impact of including multiple study reports but makes no specific recommendation [23].

Two textbooks recommend reviewers to plan in advance which data they will need to extract for their review [24, 28]. One textbook makes an optional recommendation, depending on the number of included studies [22]. For reviews with a small number of studies it considers an iterative process appropriate; for large data sets it recommends a thoroughly developed and overinclusive extraction form to avoid the need to go back to study reports later in the review process.

One textbook recommends that clinical experts or methodologists are consulted in developing the extraction form to ensure important study aspects are included [26]. None includes statements on the recording and handling of extraction errors.

#### Category: piloting of extraction forms

For this category, the most frequently made recommendation in the analysed textbooks is that reviewers should pilot test their data extraction form (8/11) [3, 20, 22–26, 29]. One textbook makes a general statement on piloting, but no specific recommendation [27].

Three textbooks recommend that data extractors are trained [22, 24, 25]. One textbook states that extraction should not begin before satisfactory agreement is achieved but does not define how this should be assessed [22]. No recommendations were identified for any of the other items regarding piloting of extraction form in the analysed textbooks.

#### Category: data extraction

Six textbooks recommend data extraction by at least two reviewers [22–26, 29]. Four of these recommend parallel extraction [23–26], while two do not specify the exact procedure [22, 29]. One textbook explains the different types of dual extraction modes but makes no recommendation on their use [27].

One textbook recommends that reviewer agreement for extracted data is quantified using a reliability measure [25], while two mention this possibility without making a clear recommendation [22, 26]. Two of these mention Cohen’s kappa as possible measures for quantifying agreement [22, 26], one also mentions raw agreement [22].

Five textbooks recommend that reviewers develop explicit procedures for resolving disagreements, either by discussion or consultation of a third person [22, 24–26, 29]. Two textbooks suggest a hierarchical approach using discussion and, if this is unsuccessful, arbitration with a third person [25, 29]. One textbook also suggests the possibility of including the entire review team in discussions [24]. One textbook emphasizes that educated discussions should be preferred over voting procedures [26]. One textbook also recommends that reviewers document disagreements and how they were resolved [26].

One textbook makes recommendations on the expertise of the data extractors [24]. It suggests that data extraction is conducted by statisticians, data managers and methods experts with the possible involvement of content experts, when required.

### Documents from HTA agencies

#### Category: development of extraction forms

In two documents from HTA agencies it is recommended that a customised extraction form is developed [31, 35]. One of these roughly outlines the contents of extraction forms that can be used as a starting point [31]. Three documents recommend that detailed instructions on using the extraction form should be provided [30, 31, 34]. Two documents recommend that reviewers develop a strategy for obtaining unpublished data [30, 31].

The following recommendations are only included in one method document each: planning in advance which data will be required for the synthesis [30]; ensuring consistent coding and response options in the data collection form [31] and linking multiple reports of the same study to avoid including data from the same study more than once [31].

#### Category: piloting of extraction forms

For this category the only recommendation we found in HTA documents is that data collection forms should be piloted before use (3/5) [30, 31, 33]. None of the documents specifies how this may be done, for example regarding the number or types of studies involved. One of the documents makes a vague suggestion that all reviewers ought to be involved in pilot testing.

#### Category: data extraction

In most documents it is recommended that data extraction should be conducted by two reviewers (4/5) [30, 31, 34, 35]. Two make an optional recommendation for either parallel extraction or a double-checking procedure [30, 31], one recommends parallel extraction [34] and one reports use of double-checking [35]. Three method documents recommend that reviewers resolving disagreements by discussion [30, 31, 35]. One method document recommends that reviewers report who was involved in data extraction [34].

### Journal articles

We identified 5 journal articles that fulfilled our inclusion criteria. This included a journal article specifying the methods used by the Cochrane Back and Neck Group [36], an article describing the data extraction and synthesis methods used in JBI systematic reviews [38], a paper on guidelines for systematic review in the environmental research field [39] and two in-depth papers on data extraction and coding methods within systematic reviews [37, 40]. One of these used the Systematic Reviews Data Suppository (SRDS) as an example, but the recommendations made were not exclusive to this system [37].

#### Category: development of extraction forms

Three journal articles recommended that authors should plan in advance which data they require for the review [37, 39, 40]. A recommendation for developing a customized extraction form (or adapting one) for the specific purpose of the review was also made in three journal articles [36, 37, 40]. Two articles recommended that consistent and clear coding and response options should be ensured and detailed instruction provided to data extractors [37, 40]. Furthermore, two articles recommended that mechanisms should be in place for recording, assessing and correcting data entry errors [36, 37]. Both referred to plausibility or logic checks of the data and/or statistics.

One article recommends that reviewers try to obtain further data from the included studies, where required [39], while one makes an optional recommendation [36] and another a general statement without a specific recommendation [37]. One of the articles also makes recommendations on the expertise of the reviewers that should be involved in the development of the extraction form. It recommends that all members of the team are involved including data extractors, content area experts, statisticians and reviewers with formal training in form design such as epidemiologists [37].

#### Category: piloting of extraction forms

Four articles recommend that reviewers should pilot test their extraction form [36–38, 40]. Three articles recommend training of data extractors [37, 38, 40]. One recommends that reviewers informally assess the reliability of coding during the piloting process [37]. One article mentions the possibility of quantifying agreement during the piloting process, without making a specific recommendation or specifying any thresholds [40].

#### Category: data extraction

Three articles recommend that data are extracted by two reviewers, in each case using independent parallel extraction [36–38]. Citing the IoM standards, one article also mentions the possibility of a using independent parallel extraction for critical data and a double-checking procedure for non-critical data [37]. One article recommends that the principle reviewer runs regular logic checks to validate the extracted data [37]. One article also mentions the possibility that the reliability of extraction may need to be reviewed throughout the extraction process in case of extended coding periods [40].

Two articles mention the need to have a procedure in place for resolving disagreements, either with a hierarchical procedure using discussion and arbitration with a third person [36] or by discussion and review of the source document [37]. One article recommends that disagreements and consensus results are documented for future reference [37]. Finally, one article mentions advantages of having data extractors with complementary expertise such as a content expert and method experts, but does not make a clear recommendations on this [37].

## Discussion

We reviewed current recommendations on data extraction methods in systematic reviews across a different range of sources. Our results suggest that current recommendations are fragmented. Very few documents made comprehensive recommendations. This may be detrimental to the quality of systematic reviews and makes it difficult to aspiring reviewers to prepare high quality data extraction forms and ensure reliable and valid extraction procedures. While our review cannot show that improved recommendations will truly have an impact on the quality of systematic reviews, it seems reasonable to assume that clear and comprehensive recommendations are a prerequisite to high quality data extraction, especially for less experienced reviewers.

There were some notable exceptions to our findings. Among the most comprehensive documents were the Cochrane Handbook for Systematic Reviews, the textbook by Foster and colleagues and the journal article by Li and colleagues [18, 24, 37]. We believe that these are among the most helpful resources for systematic reviewers from the pool of documents that we analysed – not only because they provide in-depth information, but also for being among the most current sources.

We were particularly surprised by the lack of information provided by HTA agencies. Only very few HTA agencies had documents with relevant recommendations at all. Since many HTA agencies publish detailed documents on many other methodological aspects such as search screening methods, risk of bias assessments or evidence grading methods, it would seem reasonable to provide more information on data extraction methods.

We believe there would be many practical benefits of developing clearer recommendations for the development and testing of extraction forms and the data extraction process. One reason is that data extraction is one of the most resource intensive parts of a systematic review – especially, when the review includes a significant number of studies and/or outcomes. Having a good extraction form can also save time at later stages of the review. For example, a poorly developed extraction form may lead to extensive revisions during the review process and may require reviewers to go back to the original sources or repeat extraction on some included studies. Furthermore, some methodological standards such as independent parallel extraction could be modified to save resources. This is not reflected in most of the sources included in our review. Lastly, it would be helpful to specify recommendations further to accommodate for systematic reviews of different sizes, both in terms of the number of included studies and the review team. While the general quality standards should remain the same, a mega-review with several tens or even hundreds of studies, a large, heterogeneous or international review team and several data extractors may differ in some requirements from a small review with few studies and a small, local team [12, 37]. For example, training and piloting may need more time to achieve agreement. We therefore encourage developers of guidelines documents for systematic reviews to provide more comprehensive recommendations on developing and piloting data extraction forms and the data extraction process. Our review can be used as a starting point. Formal development of structured guidance or a set of minimum standards on data extraction methods in systematic reviews may also be useful. Moher and colleagues have developed a framework to support the development of guidance to improve reporting, which includes literature reviews and a Delphi study and provides a helpful starting point [41]. Lastly, authors of reporting guidelines for systematic reviews of various types can use our results to consider elements worth including.

To some extent the results reflect the empirical evidence from comparative methods research. For example, among the most frequent recommendations were that data extraction should be conducted by two reviewers to reduce risk of errors, which is supported by some evidence [11]. This is also true for the recommendation that additional data should be retrieved if necessary, which reflects selective outcome reporting [42]. At the same time, we found few recommendations on reviewer expertise, for which empirical studies have produced inconsistent results [11]. Arguably, some items in our analysis have theoretical rather than empirical foundations. For instance, we would consider the inclusion of content experts in the development of the extraction forms to be important to enhance clinical relevance and applicability. Even this is a somewhat contested issue, however. Gøtzsche and Ioannidis, for instance, have questioned the value of involving content experts in systematic reviews [43]. In their analysis, they highlight the lack of evidence on the effects of involving them and in addition to the possible benefits raise potential downsides of expert involvement – notably that experts often have conflicts of interest and strong prior opinions that may introduce bias. While we do not argue against involvement of content experts since conflicts of interest can be managed, the controversy shows that this in fact may be an issue worth exploring empirically [44]. Thus, in addition to providing more in-depth recommendations for systematic reviewers, empirical evaluations of extraction methods should be encouraged. Such method studies should be based on a systematic review of the current evidence and overcome some of the limitations from previous investigations including the use of convenience samples and small sets of reviewers [11].

As a final note, some parts of systematic reviews can now be assisted by automation methods. Examples include enhanced study selection using learning algorithms (e.g. implemented in Rayyan) and assisted risk of bias assessments using RobotReviewer [45, 46]. However, not all of the software solutions are free and some are still in their early development or have not been validated yet. Furthermore, some of them are restricted to specific review types [47]. To the best of our knowledge comprehensive tools to assist with data extraction, including for example extraction of outcome data, are no yet available [48]. For example, a recent systematic review conducted with currently available automation tools used traditional spreadsheet-based data extraction forms and piloting methods [49]. The authors identified two issues regarding data extraction that could be assisted by automation methods: contacting authors of included studies for additional information using metadata and better integration of software tools to automatically exchange data between different software. Thus, much work is still to be done in this area. Furthermore, when automation tools for data extraction become available, they will need to be readily available, usability tested, accepted by systematic reviewers and validated before widespread use (validation is especially important for technically complex or critical tasks) [50]. It is also likely that they will complement current data extraction methods rather than replace them as it is currently the case for automated risk of bias assessments of randomised trials [46]. For these reasons we believe that traditional data extraction methods will still be required and used in the future.

### Limitations

There are some limitations to our methods. Firstly, our review is not exhaustive. The list of handbooks from SROs was compiled based on previous research and discussions between the authors, but no formal search was conducted to identify other potentially relevant organisations [51, 52]. The list of textbooks was also based on a previous study not intended to cover the literature in full. It does, however, include textbooks from a range of disciplines including medicine, nursing, education and the social sciences, which arguably increases the generalisability of the findings. The search strategy for our database search was pragmatic for reasons stated in the methods and may have missed some relevant articles. Furthermore, the databases searched focus on the field of medicine and health, so other areas may be underrepresented.

Secondly, searching the websites of HTA agencies proved difficult in some instances, as some websites have quite intricate site structures. Furthermore, we did not contact the HTA agencies to retrieve unpublished documents. It is likely that at least some HTA agencies have internal documents that provide more specific recommendations. Our focus was the usefulness of the HTA method documents as a guidance to systematic reviewers outside of HTA institutions, however. For this purpose, we believe that the assumption is appropriate that most reviewers are likely to depend on the information directly accessible to them.

Thirdly, it was difficult to classify some of the recommendations using our coding scheme. For example, recommendations in the new Cochrane Handbook are based on Cochrane’s Methodological Expectations for Cochrane Intervention Reviews Standards (MECIR) which make a subtle differentiation between mandatory and highly desirable recommendations. In this case we considered both these types of recommendations as positive in our classification scheme. To use a more difficult example, one HTA method document did not make a statement on the number of reviewers involved in data extraction but stated that a third investigator may check a random sample of extracted data for additional quality assurance. This would imply that data extraction is conducted by two reviewers independently, but since this method was not stated, it was classified as “method not mentioned”. While some judgements were required, we have described notable cases in the results section and do not believe that different decisions in these cases would affect our overall results or conclusions.

Lastly, we note that some of the included sources referenced more comprehensive guidance such as the Cochrane Handbook. We have not formally extracted information on cross-referencing between documents, however.

## Conclusion

Many current methodological guidance documents for systematic reviewers lack comprehensiveness and clarity regarding the development and piloting of data extraction forms and the data extraction process. In the future, developers of learning resources should consider providing more information and guidance on this important part of the systematic review process. Our review and list of items may be a helpful starting point. HTA agencies may consider describing in more detail their published methods on data extraction procedures to increase transparency.

## Supplementary information

**Additional file 1.** List of HTA websites searched.

**Additional file 2.** Information on database searches

**Additional file 3.** List of items and rationale

**Additional file 4.** List of included documents

**Additional file 5.** Recommendations for non-interventional reviews

**Additional file 6.** Primary analysis

## Data Availability

The datasets used and analysed for the current study are available from the corresponding author on reasonable request.

## Abbreviations

CMR: Cochrane Methodology Register
CRD: Centre for Reviews and Dissemination
EUnetHTA: European Network for Health Technology Assessment
HTA: Health Technology Assessment
HTAi: Health Technology Assessment international
HTAsiaLink: The collaborative research network of Health Technology Assessment agencies in the Asia-Pacific region
INAHTA: International Network of Agencies for Health Technology Assessment
IoM: Institute of Medicine
JBI: Joanna Briggs Institute
PRIMSA: Preferred Reporting Items for Systematic Reviews and Meta-Analyses
RedETSA: Red de Evaluación de Tecnologías en Salud de las Américas (Health Technology Assessment Network of the Americas)
SRCML: Scientific Resource Center’s Methods Library
SROs: Systematic Review Organisations
