# Supplementary material for: Development, testing and use of data extraction forms in systematic reviews: a review of methodological guidance
Source: BMC Med Res Methodol. 2020 Oct 19;20:259. doi: 10.1186/s12874-020-01143-3 (PMC7574308; doi:10.1186/s12874-020-01143-3)
Supplement: Supplementary file 1 — Additional file 1. List of HTA websites searched. [file 12874_2020_1143_MOESM1_ESM.docx]

**Additional file 1: HTA agencies websites searched for relevant documents**

| **No.** | **Institution** | **Country/Umbrella Institution(s)** | **URL** |
| --- | --- | --- | --- |
|  | [AAZ](https://www.eunethta.eu/aaz) Agency for Quality and Accreditation in Health Care and Social Welfare | Croatia/EUnetHTA | <http://www.aaz.hr/> |
|  | **ACE** **Agency for Care Effectiveness** | Singapore/INAHTA | <http://www.ace-hta.gov.sg/> |
|  | [ACSS IP](https://www.eunethta.eu/acss-ip) Administração Central do Sistema de Saúde, I.P. | Portugal/EUnetHTA | <http://www.acss.min-saude.pt/> |
|  | [AEMPS](https://www.eunethta.eu/aemps) Agencia Española de Medicamentos y Productos Sanitarios | Spain/EUnetHTA+HTAi | [https://www.aemps.gob.es](https://www.aemps.gob.es/) |
|  | **AETS** **Agencia de Evaluación de Tecnologias Sanitarias** | Spain/INAHTA | <http://www.isciii.es/ISCIII/es/contenidos/fd-el-instituto/fd-organizacion/fd-estructura-directiva/fd-subdireccion-general-programas-internacionales-investigacion-relaciones-institucionales/fd-centros-unidades/agencia-evaluacion-tecnologias-sanitarias.shtml> |
|  | [AETSA](https://www.eunethta.eu/aetsa) Andalusian HTA Agency | Spain/EUnetHTA+HTAi+INAHTA | <http://www.aetsa.org/> |
|  | [AETS-ISCIII](https://www.eunethta.eu/aets-isciii) The Instituto De Salud Carlos III | Spain/EUnetHTA | [http://www.eng.isciii.es](http://www.eng.isciii.es/) |
|  | [Agenas](https://www.eunethta.eu/agenas) National Agency for Regional Health Services | Italy/EUnetHTA+HTAi+INAHTA | [http://www.agenas.it](http://www.agenas.it/)  [agenas@pec.agenas.it](mailto:agenas@pec.agenas.it) |
|  | **AHRQ** **Agency for Healthcare Research and Quality** | USA/HTAi+INAHTA | [http://www.ahrq.gov](http://www.ahrq.gov/) |
|  | **AHTA** **Adelaide Health Technology Assessment** | Australia/INAHTA | [http://www.adelaide.edu.au/ahta](http://www.adelaide.edu.au/ahta/) |
|  | **AHTAPol** **Agency for Health Technology Assessment in Poland** | Poland/INAHTA | [http://www.aotm.gov.pl](http://www.aotm.gov.pl/) |
|  | [AIFA](https://www.eunethta.eu/aifa) Italian Medicines Agency | Italy/EUnetHTA+HTAi | [http://www.agenziafarmaco.gov.it](http://www.agenziafarmaco.gov.it/) |
|  | **Alberta Health Services** | Canada/HTAi | <https://www.albertahealthservices.ca/> |
|  | **ANVISA** **National Health Surveillance Agency** | Brazil/RedETSA | <https://www.emergobyul.com/de/resources/brazil/anvisa>  http://portal.anvisa.gov.br/ |
|  | [AOTMiT](https://www.eunethta.eu/aotmit) Agency for Health Technology Assessment and Tariff System | Poland/EUnetHTA | [http://www.aotmit.gov.pl](http://www.aotmit.gov.pl/) |
|  | [AQuAS](https://www.eunethta.eu/aquas) Agency for Health Quality and Assessment of Catalonia | Spain/EUnetHTA+HTAi+INAHTA | <http://aquas.gencat.cat/ca/inici/> |
|  | **Argentinean Ministry of Health** | Argentina/RedETSA | <https://www.argentina.gob.ar/salud> |
|  | **ASERNIP-S** **Australian Safety and Efficacy Register of New Interventional Procedures – Surgical** | Australia/INAHTA | [http://www.surgeons.org/racs/research-and-audit/asernip-s](http://http/www.surgeons.org/racs/research-and-audit/asernip-s) |
|  | **ASSR** **Agenzia Sanitaria e Sociale Regionale (Regional Agency for Health and Social Care)** | Italy/INAHTA | <http://assr.regione.emilia-romagna.it/it> |
|  | [AVALIA FNS](https://www.eunethta.eu/avalia-fns) Fundacion Profesor Novoa Santos | Spain/EUnetHTA | <http://hospitalcoruna.sergas.es/investigacion/fundacion/Paginas/inicio.aspx>  http://www.fundacionprofesornovoasantos.org/es/ |
|  | [AVALIA-T](https://www.eunethta.eu/avalia-t) Galician Agency for HTA | Spain/EUnetHTA+HTAi+INAHTA | <http://avalia-t.sergas.es> |
|  | [AWTTC](https://www.eunethta.eu/awttc) All Wales Therapeutics and Toxicology Centre | United Kingdom/EUnetHTA | [http://awttc.org](http://awttc.org/) |
|  | BAG **(Bundesamt für Gesundheit)/**FOPH **(Federal Office of Public Health)** | Switzerland | <https://www.bag.admin.ch/bag/de/home.html> |
|  | **BCBSA** **Blue Cross Blue Shield Association** | USA/HTAi | <https://www.bcbs.com/> |
|  | [BIOEF](https://www.eunethta.eu/bioef) Basque Foundation for Health Innovation and Research | Spain/EUnetHTA | <http://www.bioef.org/> |
|  | **Bolivian Ministry of Health** | Bolivia/RedETSA | <https://www.minsalud.gob.bo/> |
|  | **Brazilian Ministry of Health** | Brazil/HTAi | <http://portalms.saude.gov.br/> |
|  | **CADTH** **Canadian Agency for Drugs and Technologies in Health** | Canada/HTAi+INAHTA | [http://www.cadth.ca](http://www.cadth.ca/) |
|  | **CCATES** **The Collaborating Center of the SUS for Health Technology Assessment and Excellence/Federal University of Minas Gerais (UFMG)** | Brazil/RedETSA | <http://www.ccates.org.br/> |
|  | **CCSS** **Costa Rican Social Security Fund** | Costa Rica/RedETSA | <https://www.ssa.gov/policy/docs/progdesc/ssptw/2014-2015/americas/costa-rica.html>  https://www.ccss.sa.cr/ |
|  | CDE **Center for Drug Evaluation, Taiwan** | Republic of China/INAHTA | [http://www.cde.org.tw](http://www.cde.org.tw/) |
|  | **CEDIT** **Comité d’Evaluation et de Diffusion des Innovations Technologiques** | France/INAHTA | [http://cedit.aphp.fr](http://cedit.aphp.fr/) |
|  | **CEEBM VMH-FMUI** Unit Clinical Epidemiology & Evidence-based Medicine, Faculty of Medicine University of Indonesia | Indonesia/HTAi | <http://www.ceebm.org/> |
|  | **CEM** **Inspection Générale de la sécurité sociale (IGSS), Cellule d’expertise médicale** | Luxembourg/INAHTA | [http://www.mss.public.lu/acteurs/igss/cem/index.html](http://http/www.mss.public.lu/acteurs/igss/cem/index.html) |
|  | **CENETEC** **Centro Nacional de Excelencia Tecnológica en Salud**  **National Center for Technological Excellence in Health, PAHO/WHO Collaborating center** | Mexico/HTAi+INAHTA/RedETSA | [http://www.cenetec.salud.gob.mx](http://www.cenetec.salud.gob.mx/) |
|  | **CGATS/MS** **Health Technology Assessment Coordination Office of the Ministry of Health** | Brazil/RedETSA | <http://portalms.saude.gov.br/> |
|  | **Changi General Hospital** | Singapore/HTAi | <https://www.cgh.com.sg/Pages/Home.aspx> |
|  | [CHIF](https://www.eunethta.eu/chif) Croatian Health Insurance Fund | Croatia/EUnetHTA | [www.hzzo.hr](http://www.hzzo.hr/) |
|  | **Chilean Ministry of Health** | Chile/RedETSA | <https://www.gob.cl/en/ministries/ministry-of-health/>  https://www.minsal.cl/ |
|  | [CIPH](https://www.eunethta.eu/ciph) Croatian Institute of Public Health | Croatia/EUnetHTA | <http://www.hzjz.hr/english/> |
|  | **CMeRC** **Charlotte Maxeke Medical Research Cluster** | South Africa/HTAi+INAHTA | [http://www.cmerc.org](http://www.cmerc.org/) |
|  | **CMTP** **Center for Medical Technology Policy** | USA/HTAi | <http://www.cmtpnet.org/> |
|  | **Colombian Ministry of Health and Social Protection** | Colombia/RedETSA | <https://www.minsalud.gov.co/English/Paginas/inicio.aspx> |
|  | **CONITEC** **National Committee for Incorporation of Technology (in the Unified Health System (SUS))** | Brazil/HTAi+INAHTA/RedETSA | [http://www.conitec.gov.br/](http://conitec.gov.br/)  http://conitec.gov.br/en/ |
|  | [CRUF/AOUIVR](https://www.eunethta.eu/cruf-aouivr) Centro Regionale Unico sul Farmaca del Veneta | Italy/EUnetHTA+INAHTA | http://www.ospedaleuniverona.it/ecm/home |
|  | **Cuban Ministry of Health** | Cuba/RedETSA | <http://www.sld.cu/> |
|  | **CUFAR** **University Center for Pharmacology of the National University of La Plata** | Argentina/RedETSA | <http://www.med.unlp.edu.ar/centros/cufar/> |
|  | **Danish Health Authority** | Denmark/HTAi | <https://www.sst.dk/en> |
|  | **DECIT-CFGATS** Coordenação Geral de Avaliação de Tecnologias em Saúde; Departamento de Ciência e Tecnologia | Brazil/INAHTA | <http://portal.saude.gov.br/portal/saude/area.cfm?id_area=1026> |
|  | [DEFACTUM (formerly CFK)](https://www.eunethta.eu/defactum-formerly-cfk) **Social & Health Services and Labour MArket** | Denmark/EUnetHTA | <http://www.defactum.net/> |
|  | [DGFDM IT](https://www.eunethta.eu/dgfdm-it) Sede del Ministro – Ministero della salute | Italy/EUnetHTA | <http://www.salute.gov.it/> |
|  | [DGFPS MSPSI](https://www.eunethta.eu/dgfps-mspsi) Directorate General for Pharmacy and Health Care Products | Spain/EUnetHTA | [DGFPS MSPSI](https://www.eunethta.eu/dgfps-mspsi)  https://www.mscbs.gob.es/profesionales/farmacia/organizacion.htm |
|  | [DIMDI](https://www.eunethta.eu/dimdi) German Institute for Medical Documentation and Information | Germany/EUnetHTA | [http://www.dimdi.de/](http://www.dimdi.de/static/de/index.html) |
|  | **Directorate of Scientific and Technological Development in Health/Ministry of Health** | Costa Rica/RedETSA | <https://www.ministeriodesalud.go.cr/> |
|  | [DPA/MoH Malta](https://www.eunethta.eu/dpa-moh-malta) Directorate for Pharmaceutical Affairs | Malta/EUnetHTA | <https://health.gov.mt/en/pharmaceutical/Pages/pharmaceutical-affairs.aspx> |
|  | **Ecuadorian Ministry of Health** | Ecuador/RedETSA | <https://www.salud.gob.ec/> |
|  | **Effective Basic Services** | Africa/HTAi | <https://ebaseafrica.wordpress.com/> |
|  | **EFPIA** | Belgium/HTAi | <https://efpia.eu/> |
|  | [EKAPTY SA](https://www.eunethta.eu/ekapty-sa) National Evalution Center of Quality and Technology in S.A.- EKAPTY | Greece/EUnetHTA | <https://www.ekapty.gr/> |
|  | [EKAPTY-NKUA](https://www.eunethta.eu/nkua) National and Kapodistrian University of Athens | Greece/EUnetHTA | <http://www.phs.uoa.gr/> |
|  | **El Salvadoran Ministry of Health** | El Salvador/RedETSA | <http://www.salud.gob.sv/> |
|  | [EOF](https://www.eunethta.eu/eof) National Organization for Medicines | Greece/EUnetHTA | [http://www.eof.gr](http://www.eof.gr/) |
|  | [EOPYY](https://www.eunethta.eu/eopyy) National Organisation for Healthcare Provision | Greece/EUnetHTA | [http://www.eopyy.gov.gr](http://www.eopyy.gov.gr/) |
|  | [EUR](https://www.eunethta.eu/eur) Erasmus Universiteit Rotterdam | Netherlands/EUnetHTA | <http://www.eur.nl/> |
|  | [FIMEA](https://www.eunethta.eu/fimea) Finnish Medicines Agency | Finland/EUnetHTA | [http://www.fimea.fi](http://www.fimea.fi/) |
|  | **FinCCHTA**  **Finnish Coordinating Center for Health Technology Assessment** | Finnland/HTAi+INAHTA | [http://www.fincchta.fi](http://www.fincchta.fi/) |
|  | **FNR** **National Resources Fund** | Uruguay/RedETSA | <http://www.fnr.gub.uy/> |
|  | [FPS](https://www.eunethta.eu/fps) Fundación Pública Andaluza Progreso y Salud | Spain/EUnetHTA | <http://www.juntadeandalucia.es/fundacionprogresoysalud/> |
|  | [Funcanis](https://www.eunethta.eu/funcanis) Fundación Canaria de Investigación Sanitaria | Spain/EUnetHTA | <http://www.funcanis.org/> |
|  | [GBA](https://www.eunethta.eu/gba) Gemeinsamer Bundesausschuss (Federal Joint Comittee) | Germany/EUnetHTA+INAHTA | <http://www.g-ba.de/> |
|  | [GOeG](https://www.eunethta.eu/gog/) Gesundheit Österreich GmbH/Geschäftsbereich | Austria/EUnetHTA+INAHTA | https://goeg.at/ |
|  | **HAD-Uruguay** **Health Assessment Division, Ministry of Public Health** | Uruguay/INAHTA/RedETSA | [http://www.msp.gub.uy](http://www.msp.gub.uy/) |
|  | [HAS](https://www.eunethta.eu/has) French National Authority for Health (Haute Autorité de Santé) | France/EUnetHTA+HTAi+INAHTA | [http://www.has-sante.fr/](http://www.has-sante.fr/portail/jcms/c_5443/english?cid=c_5443) |
|  | [Hdir](https://www.eunethta.eu/hdir) Norwegian Directorate of Health | Norway/EUnetHTA | <https://helsedirektoratet.no/english> |
|  | **Health Technology Assessment Association** | Russia/HTAi | <http://eng.hta-rus.ru/> |
|  | **HTW****Health Technology Wales** | United Kingdome/HTAi/INAHTA | <http://www.healthtechnology.wales/> |
|  | **HealthPACT** **Health Policy Advisory Committee on Technology** | Australia/INAHTA | <http://www.health.qld.gov.au/healthpact/> |
|  | [HI](https://www.eunethta.eu/hi) The Institute of Hygiene | Lithania/EUnetHTA | <http://www.hi.lt/> |
|  | [HIQA](https://www.eunethta.eu/hiqa) Health Information and Quality Authority | Ireland/EUnetHTA+HTAi+INAHTA | <http://www.hiqa.ie/> |
|  | [HIS](https://www.eunethta.eu/his) Healthcare Improvement Scotland | United Kingdom/EUnetHTA+HTAi+INAHTA | [http://www.healthcareimprovementscotland.org](http://www.healthcareimprovementscotland.org/) |
|  | **HQO** **Health Quality Ontario (Evidence Development and Standards Branch)** | Canada/HTAi+INAHTA | <http://www.hqontario.ca/> |
|  | [HVB](https://www.eunethta.eu/hvb/) Hauptverband der Österreichischen Sozialversicherungsträger (Association of Austrian Social Insurance Institutions) | Austria/EUnetHTA | [http://www.sozialversicherung.at](http://www.sozialversicherung.at/) |
|  | **IACS** **Health Sciences Institute in Aragon** | Spain/INAHTA | <http://www.iacs.es/> |
|  | **ICER** **Institute for Clinical & Economic Review** | USA/HTAi | <https://icer-review.org/> |
|  | **IECS** **Institue for Clinical Effectiveness and Health Policy (PAHO/WHO Collaborating Center)** | Argentina/INAHTA/RedETSA | [http://www.iecs.org.ar](http://www.iecs.org.ar/) |
|  | **IETS** **Instituto de Evaluación Tecnológica en Salud**  **Health Technology Assessment Institute** | Colombia/INAHTA/RedETSA | [http://www.iets.org.co](http://www.iets.org.co/) |
|  | **IETSI-ESSALUD** **Institute of Health Technology Assessment and Research** | Peru/RedETSA | <http://www.essalud.gob.pe/ietsi/> |
|  | [IFET](https://www.eunethta.eu/ifet) Institute of Pharmaceutical Research and Technology | Greece/EUnetHTA | <http://www.ifet.gr/english_site/> |
|  | **IHE** **Institute for Health Economics** | Canada/HTAi+INAHTA | [http://www.ihe.ca](http://www.ihe.ca/) |
|  | **INAHTA** **Checklists for Health Technology Assessment Reports** | INAHTA | <http://www.inahta.org/> |
|  | **I**NC **National Cardiology Institute** | Brazil/RedETSA | [www.inc.saude.gov.br](http://www.inc.saude.gov.br) |
|  | **INEAS** **National Authority for Assessment and Accreditation in Healthcare** | Tunisia/INAHTA | www.ineas.tn |
|  | **INESSS** **Institut national d’excellence en santé et en services**  **National Institute for Excellence in Health and Social Services** | Canada/INAHTA/RedETSA | [http://www.inesss.qc.ca](http://www.inesss.qc.ca/) |
|  | [INFARMED](https://www.eunethta.eu/infarmed) National Authority of Medicines and Health Products | Portugal/EUnetHTA | <http://www.infarmed.pt/> |
|  | **INS** **National institute of Health** | Peru/RedETSA | <https://web.ins.gob.pe/> |
|  | **Institute of Population Health/University of Ottawa** | Canada/RedETSA | <http://www.cihr-irsc.gc.ca/e/13777.html> |
|  | [IPH](https://www.eunethta.eu/iph) Scientific Institute of Public Health | Belgium/EUnetHTA | <https://www.wiv-isp.be/en> |
|  | [IQWIG](https://www.eunethta.eu/iqwig) Institute for Quality and Efficiency in Health Care | Germany/EUnetHTA+HTAi+INAHTA | <http://www.iqwig.de/index.2.en.html> |
|  | **ISP** **Institute of Public Health** | Chile/RedETSA | <http://www.ispch.cl/> |
|  | **ISSS** **Salvadoran Social Insurance Institute** | EL Salvador/RedETSA | <http://www.isss.gob.sv/> |
|  | [JAZMP](https://www.eunethta.eu/jazmp) Public Agency of the Republic of Slovenia for Medicinal Products and Medical Devices | Slovenia/EUnetHTA | <https://www.jazmp.si/en/> |
|  | **Kaiser Permanente** | USA/HTAi | <https://healthy.kaiserpermanente.org/> |
|  | [KCE](https://www.eunethta.eu/kce) Belgian Health Care Knowledge Centre | Belgium/EUnetHTA | [https://kce.fgov.be](https://kce.fgov.be/) |
|  | [LBI-HTA](https://www.eunethta.eu/lbi/) Ludwig Boltzmann Institute for Health Technology Assessment | Austria/EUnetHTA+INAHTA+INAHTA | [http://hta.lbg.ac.at](http://hta.lbg.ac.at/) |
|  | **MaHTAS**  **Health Technology Assessment Section,**  **Ministry of Health** | Malaysia/HTAi+INAHTA | [http://www.moh.gov.my](http://www.moh.gov.my/) |
|  | [MoH Cyprus](https://www.eunethta.eu/moh-cyprus) Ministry of Health of Cyprus | Cyprus/EUnetHTA | <http://www.moh.gov.cy/moh/phs/phs.nsf/dmlindex_en/dmlindex_en?opendocument> |
|  | [MoH Czech](https://www.eunethta.eu/moh-czech) Ministry of Health of the Czech Republic | Czech Republic/EUnetHTA | <http://www.mzcr.cz/en/> |
|  | [MoH Slovak Republic](https://www.eunethta.eu/moh-slovak-republic) Ministry of Health of the Slovak Republic | Slovakia/EUnetHTA | [www.health.gov.sk](http://www.health.gov.sk/) |
|  | [MoH Slovenia](https://www.eunethta.eu/moh-slovenia) Ministry of Health of the Republic of Slovenia | Slovenia/EUnetHTA | <http://www.mz.gov.si/en/> |
|  | [MPA](https://www.eunethta.eu/mpa) Medical Products Agency | Sweden/EUnetHTA | [https://lakemedelsverket.se](https://lakemedelsverket.se/) |
|  | [NCPE](https://www.eunethta.eu/ncpe) National Centre for Pharmacoeconomics, St. James Hospital | Ireland/EUnetHTA | [http://www.ncpe.ie](http://www.ncpe.ie/) |
|  | [NCPHA](https://www.eunethta.eu/ncpha) National Center of Public Health and Analyses | Bulgaria/EUnetHTA | [http://ncpha.government.bg](http://ncpha.government.bg/) |
|  | **NECA** **National Evidence-based Healthcare Collaborating Agency** | South Korea/HTAi+INAHTA | [http://www.neca.re.kr](http://www.neca.re.kr/) |
|  | [NICE](https://www.eunethta.eu/nice) National Institute for Health and Care Excellence | United Kingdom/EUnetHTA+HTAi | <http://www.nice.org.uk/> |
|  | **NIHR** **Innovation Observatory, Newcastle University** | United Kingdom/HTAi | <http://www.io.nihr.ac.uk/> |
|  | **NIHR**  **National Institute for Health Research** | United Kingdom/INAHTA | [http://www.hta.ac.uk](http://www.hta.ac.uk/) or <http://www.nihr.ac.uk/funding/health-technology-assessment.htm> |
|  | [NIJZ](https://www.eunethta.eu/nijz) National instute of Public Health (NIJZ) | Slovenia/EUnetHTA | <http://www.nijz.si/en> |
|  | [NIPHB](https://www.eunethta.eu/niphb) Institutu National De Sanatate Publica (INSP) | Romania/EUnetHTA | [http://www.insp.gov.ro](http://www.insp.gov.ro/) |
|  | [NIPHNO (formerly NOKC)](https://www.eunethta.eu/niphno-formerly-nokc) The Norwegian Institute of Public Health | Norway/EUnetHTA+HTAi | [http://www.fhi.no/](http://www.nokc.no/) |
|  | [NIPN](https://www.eunethta.eu/nipn) National Institute of Pharmacy and Nutrition | Hungary/EUnetHTA | <https://www.ogyei.gov.hu/main_page/> |
|  | [NOMA](https://www.eunethta.eu/noma) Norwegian Medicines Agency | Norway/EUnetHTA | [www.legemiddelverket.no](http://www.legemiddelverket.no) |
|  | **Norwegian Centre for E-health Research** | Norway/HTAi | <https://ehealthresearch.no/en/> |
|  | **NPS**  **MedicineWise** | Australia/HTAi | <https://nps.org.au/> |
|  | [NSPHMPDB](https://www.eunethta.eu/nsphmpdb) National School of Public Health, Management and Professional Development | Romania/EUnetHTA | [http://www.snspms.ro](http://www.snspms.ro/) |
|  | [NVD](https://www.eunethta.eu/nvd) National Health Service | Latvia/EUnetHTA | <http://www.vmnvd.gov.lv/> |
|  | [OCSC](https://www.eunethta.eu/ocsc) Onassis Cardiac Surgery Centre | Greece/EUnetHTA | <http://www.onasseio.gr/> |
|  | [Osteba](https://www.eunethta.eu/osteba) Basque Office for Health Technology Assessment- Ministry for Health | Spain/EUnetHTA+HTAi+INAHTA | <http://www.osakidetza.euskadi.eus/> |
|  | **Oswaldo Cruz Foundation** | Brazil/HTAi | <https://portal.fiocruz.br/en> |
|  | **Panamanian Ministry of Health** | Panama/RedETSA | <http://www.minsa.gob.pa/> |
|  | **Paraguayan Ministry of Public Health and Social Welfare** | Paraguay/RedETSA | <https://www.mspbs.gov.py/portal> |
|  | **PBAC&MSAC** **Australia Government, Department of Health and Ageing** | Australia/HTAi | <http://www.health.gov.au/> |
|  | **PCORI** **Patient-Centered Outcomes Research Institute** | USA/HTAi | <https://www.pcori.org/> |
|  | **Peruvian Ministry of Health** | Peru/RedETSA | <http://www.minsa.gob.pe/> |
|  | **RCHD** **Republican Center for Health Development** | Kazakhstan/HTAi+INAHTA | [http://www.rcrz.kz](http://www.rcrz.kz/) |
|  | **RedArets** **Argentina Public Network of Health Technology Assessment** | Argentina/RedETSA | <http://www.redarets.com.ar/index.php/english> |
|  | [RER](https://www.eunethta.eu/rer) Regione Emilia-Romagna | Italy/EUnetHTA | <http://www.regione.emilia-romagna.it/> |
|  | [RIZIV-INAMI](https://www.eunethta.eu/riziv-inami) Rijksinstituut voor Ziekte- en Invaliditeitsverzekering | Belgium/EUnetHTA | [http://www.inami.fgov.be/](http://www.inami.fgov.be/homefr.htm) |
|  | [SBU](https://www.eunethta.eu/sbu) Swedish Agency for Health Technology Assessment and Assessment of Social Services | Sweden/EUnetHTA+HTAi+INAHTA | [www.sbu.se](http://www.sbu.se/) |
|  | [SESCS](https://www.eunethta.eu/sescs) Evaluation AND Planning Unit – Directorate of the Canary Islands Health Service | Spain/EUnetHTA | [www.sescs.es](http://www.sescs.es/) |
|  | **SFOPH** **Swiss Federal Office of Public Health** | Switzerland/HTAi | <https://www.bag.admin.ch/bag/en/home.html> |
|  | **SIS** **Comprehensive Health Insurance** | Peru/RedETSA | http://documents.worldbank.org/curated/en/371851468086931725/Perus-comprehensive-health-insurance-and-new-challenges-for-universal-coverage |
|  | [SNHTA](https://www.eunethta.eu/snhta) Swiss Network for HTA | Switzerland/EUnetHTA | <http://www.snhta.ch/> |
|  | [SU](https://www.eunethta.eu/su) Health Services Management Training Center | Hungary/EunetHTA | <http://semmelweis.hu/emk/en/> |
|  | [SUKL](https://www.eunethta.eu/sukl) State Institute for Drug Control | Czech Republic/EUnetHTA | <http://www.sukl.eu/> |
|  | [THL](https://www.eunethta.eu/thl) National Institute for Health and Welfare | Finland/EUnetHTA | <http://www.thl.fi/> |
|  | [TLV](https://www.eunethta.eu/tlv) Dental and Pharmaceutical Benefits Agency | Sweden/EUnetHTA+HTAi | [www.tlv.se](http://www.tlv.se/) |
|  | [UBB](https://www.eunethta.eu/ubb) Babes-bolayi University, Cluj School of Public Health | Romania/EUnetHTA | <http://publichealth.ro/> |
|  | [UCSC GEMELLI](https://www.eunethta.eu/ucsc-gemelli) University Hospital A. Gemelli | Italy/EUnetHTA | <http://roma.unicatt.it/> |
|  | [UMIT](https://www.eunethta.eu/umit) University for Health Sciences, Medical Informatics and Technology | Austria/EUnetHTA | [http://www.umit.at](http://www.umit.at/) |
|  | [UniBA FOF](https://www.eunethta.eu/uniba-fof) Comenius University in Bratislava | Slovakia/EUnetHTA | <https://www.fpharm.uniba.sk/en/divisions/the-faculty-pharmacy/>  https://www.fpharm.uniba.sk/en/science-and-research/ |
|  | [UTA](https://www.eunethta.eu/uta) Institute of Family Medicine and Public Health | Estonia/EUnetHTA | [www.tervis.ut.ee](http://www.tervis.ut.ee/) |
|  | [UU](https://www.eunethta.eu/uu) Utrecht University | Netherlands/EUnetHTA | [www.uu.nl](http://www.uu.nl/) |
|  | [UVTA/AOP](https://www.eunethta.eu/crehta-aop)  Unita di Valutazione Technology Assessment | Italy/EUnetHTA+INAHTA | [www.sanita.padova.it](http://www.sanita.padova.it) |
|  | [VASPVT](https://www.eunethta.eu/vaspvt) State Health Care Accreditation Agency | Lithania/EUnetHTA | [www.vaspvt.gov.lt](http://www.vaspvt.gov.lt/) |
|  | VATAP (VA Technology Assessment Program) | USA | <https://www.va.gov/vatap/> |
|  | [Veneto/CRUF](https://www.eunethta.eu/veneto-cruf) Regione Del Veneto – Area Sanita E’ Sociale | Italy/EUnetHTA | <http://www.regione.veneto.it/> |
|  | [ZIN](https://www.eunethta.eu/zin) National Health Care Institute | Netherlands/EUnetHTA+INAHTA+HTAi | <http://www.zorginstituutnederland.nl/> |
|  | **ZonMw** **The Netherlands Organisation for Health Research and Development** | Netherlands/INAHTA | [http://www.zonmw.nl](http://www.zonmw.nl/) |

**Flow Diagram for selection of relevant HTA method documents**

Full-text articles excluded (n=149)

Not a method document (n=55)

No recommendations on extraction methods (n= 39)

Language (n=46)

Outdated version (n=4)

Duplicate (n=5)

Method documents included in synthesis
(n = 6)

HTA method documents retrieved from websites
(n = 155)
