# Supplementary material for: Development, testing and use of data extraction forms in systematic reviews: a review of methodological guidance
Source: BMC Med Res Methodol. 2020 Oct 19;20:259. doi: 10.1186/s12874-020-01143-3 (PMC7574308; doi:10.1186/s12874-020-01143-3)
Supplement: Supplementary file 2 — Additional file 2. Information on database searches [file 12874_2020_1143_MOESM2_ESM.docx]

**Additional file 2: information on database searches**

**Inclusion and exclusion criteria for search results from bibliographic database searches**

| **Inclusion criteria** | **Exclusion criteria** |
| --- | --- |
| Articles including recommendations on the development, piloting and/or application of data extraction sheets for authors of systematic reviews | Introductory articles (“primers”) written for readers and users of systematic reviews such as clinicians |
| Articles published in a peer reviewed journal | Articles describing the development of extraction sheets of specific institutions (not specifically making recommendations for other reviewers) |
| Articles published in English or German | Empirical articles (e.g. analysing the broader methods used in systematic reviews or recommended) |
| Most current version in case of articles with one or more updates | Older iterations of articles with one or more updates |

**Search strategies for literature search and PRISMA flow chart**

**Medline search string (via PubMed)**

#1 "Data Collection/methods"[Mesh]

#2 "Information Storage and Retrieval/methods"[Mesh]

#3 "Information Storage and Retrieval/standards"[Mesh]

#4 "Information Storage and Retrieval/statistics and numerical data"[Mesh]

#5 “data handling”[tiab] OR (handling[tiab] AND data[tiab]) OR “data extraction”[tiab] OR “extracting data”[tiab] OR “extraction data”[tiab] OR "data abstraction"[tiab] OR “abstracting data”[tiab] OR “abstraction data”[tiab]

#6 #1 OR #2 OR #3 OR #4 OR #5

#7 "Review Literature as Topic"[Mesh]

#8 "Systematic Reviews as Topic"[Mesh]

#9 "Meta-Analysis as Topic"[Mesh]

#10 meta-analy*[tiab] OR metaanaly*[tiab] OR "meta analy*"[tiab] OR "systematic review*"[tiab] OR "literature review"[tiab]

#11 #7 OR #8 OR #9 OR #10

#12 #6 AND #11

#13 pilot*[tiab] OR recommend*[tiab] OR standard*[tiab] OR test*[tiab]

#14 #12 AND #13

#15 #14 AND ("2017/10/01"[dp]:"3000"[dp])

**Search terms used to search the Scientific Resource Center’s Methods Library (SRCML)**

Search terms used for the search field of the archived refworks library: abstraction, extraction, “data handling”, “extracting data”, “data collection”, “data entry”

**Search terms used to search the Cochrane Methodology Register (CMR)**

Title search: abstraction, extraction, data AND handling, extracting AND data, data AND collection

CMR keyword search: “data entry”

**Flow Diagram for selection of references from database search**

Records identified through Medline via PubMed
(n = 2163)

Records identified through SRCML and CMR
(n = 819)

Records screened
(n = 2982)

Records excluded
(n = 2967)

Full-text articles excluded (n=10)

Description of extraction sheet of specific institutions (n=3)

No recommendations on development, piloting or application of extraction sheets (n= 4)

Older version of an updated article (n=2)

Empirical articles (n=1)

Studies included in qualitative synthesis
(n = 5)

Full-text articles assessed for eligibility
(n = 15)
