# Supplementary material for: Development, testing and use of data extraction forms in systematic reviews: a review of methodological guidance
Source: BMC Med Res Methodol. 2020 Oct 19;20:259. doi: 10.1186/s12874-020-01143-3 (PMC7574308; doi:10.1186/s12874-020-01143-3)
Supplement: Supplementary file 3 — Additional file 3. List of items and rationale [file 12874_2020_1143_MOESM3_ESM.doc]

**Additional file 3: list of items of interest and rationale**

The following tables provide a rationale for the items we chose for analysis. The list of items was developed through iterative reading of the most influential method handbooks from SROs (see manuscript for list) and our personal experience. As there is limited empirical evidence to guide the choice of items and some items have a theoretical rather thank empirical rationale, we were inclusive when compiling this list. While the empirical research was not the scope of our paper, we referenced relevant systematic reviews or methodological studies that we are aware of.

**Dimension: Developing data extraction forms**

| Item | Rationale for inclusion in our analysis |
| --- | --- |
| Plan in advance which data are needed for the evidence synthesis | An incomplete extraction form can lead to omissions at later stages of the review or make it necessary to go back to primary studies after completion of the data collection process, possibly increasing the risk of errors. This can also be unnecessarily time-consuming. Incomplete forms may also result in less informative reviews. On the other side, unnecessary detailed data extraction sheets may also lead to resource waste. |
| Develop a customized extraction form or adapt an existing or generic form to meet the specific requirements of the specific review | There are advantages and disadvantages to using customized vs. generic extraction form. A well piloted, generic form may increase consistency, especially when used repeatedly by the same group of reviewers. A customized form increases the chances that no data are missed that are important to the requirements of the specific review. Adaptations of existing or generic forms may be an efficient compromise. The appropriate choice likely depends on different factors such as the question and type of review and the included study designs. |
| Use an existing or generic data extraction form |
| Ensure consistent and straightforward coding and responses/response options in data collection form | Coding should be as unambiguous as possible to increase reliability and reduce the risk of errors. This is especially relevant to predefined (closed) response options. |
| Provide detailed instructions on how to fill in the data collection form | Detailed instructions should increase intra-rater reliability. For example, judgment of a specific risk of bias domain depends on how well the criteria for assessing it are defined and described. Depending on the size and time schedule of a review, reviewers may extract data over a longer period with breaks between extraction of individual studies. A larger number of data extractors might also decrease consistency if coding instructions are insufficient. |
| Involve reviewers with complementary expertise in the development of the extraction form (e.g. clinical and methodological) | Reviewers need to extract data on a variety of different aspects including content specific data (e.g. characteristics on sample, intervention/exposure, setting), methodological characteristics and statistical/outcome data. Thus, including reviewers with a broad range of expertise in the development of extraction forms may be important to the relevance and quality of the form. |
| Involve reviewers with experience in systematic review methods in the development of the extraction form | Risk of bias analysis and synthesis of the primary study data are central elements of the systematic review process, suggesting that it is important to include reviewers with specific methodological expertise into the development of the extraction form. |
| Link multiple reports of the same study to avoid duplicate/ multiple inclusion(s) of the same study | Duplicate or multiple publications of the same studies are common (in one analysis they occurred in 40% of reviews) [1]. If the same data are included more than once in an analysis, this can inflate or dilute results [2]. Only reviewing one study report, when multiple publications are available risks missing important data (e.g. when publication of results is split between different publications or when non-outcome data are differentially reported between publications). |
| Develop mechanism for recording, assessing and correcting data entry errors | Analysing reasons for extraction errors that occur during the review process can improve data quality and raise discussions about possible misunderstanding or other sources of error. Keeping a paper trail of the data flow also increases transparency and ensures that possible errors can be detected and corrected at later stages of the review or post-publication. |
| Develop a strategy for obtaining unpublished data | Poor reporting and selective outcome reporting are well-known problems in systematic reviews [3]. Developing strategies to obtain missing data, for example by contacting authors, increases the quality, usefulness and validity of a systematic review. |

**Dimension: Piloting data extraction forms**

| Item | Rationale for inclusion in our analysis |
| --- | --- |
| Train data extractors in how to fill in the data collection form | In addition to providing clear instructions (e.g. by including precisely phrased coding responses and instructions or providing a coding book) training of data extractors is aimed at increasing reliability. This may be of particular importance for data extractors not involved in the development of the extraction form. |
| Pilot test data collection form using a sample of studies | Piloting data extraction forms aims to reduce the risk that important items are missed and helps to identify coding difficulties, hereby increasing consistency of extraction across study reports. |
| (Partially) repeat pilot testing if major changes are made to the data collection form during the review process | Sometimes, especially in complex reviews, it can become necessary to revise the extraction form during the review process. In this case it may be reasonable to repeat piloting for the reasons stated above. |
| In case of modifications to the data collection form, re-check reports that have already undergone data extraction | If the included studies are used to pilot the data extraction form and changes are made to the form during the piloting process, it seems prudent to re-check the reports that have already been extracted. The same is true of studies extracted earlier during the extraction process when the form is changed at later stages. |
| Involve reviewers with complementary expertise in piloting of the data collection form | For the same reason as mentioned under development of the extraction form, it seems sensible to include reviewers with complementary expertise in the piloting process. This may help to identify more specific problems with coding instructions. For example, methodologists or statisticians would be particularly well suited to prevent errors regarding methodological or statistical characteristics. This could help less experienced reviewer to avoid mistakes such as confusing standard errors and standard deviations or adequately assessing blinding of different trial personnel, for example. The same is true for the involvement of content experts for specific clinical aspects. |
| Involve reviewer with experience in systematic review methods in piloting the data collection form |
| Quantify agreement using a reliability measure such as Cohen's kappa | Agreement can be quantified by measures such as raw agreement, Cohen’s kappa or other reliability measures. There is debate about the value of using statistical measures of agreement and how it is used. Measuring agreement on data extracted from a sample of studies during piloting of the extraction form is a way of highlighting possible problems with reliability. |
| Quantify agreement using a reliability measure (e.g. Cohen's kappa) only for critical items such as risk of bias items or outcome data | Given the additional work required in quantifying agreement and the unclear value, it is sometimes recommended that if this is done, it should be restricted to important extraction items. |
| Repeat the piloting process until a specified agreement is reached such as Cohen's Kappa > 0.7 or agreement > 80% | Some reviewers pilot extraction forms until a specified threshold on a reliability measure is achieved. |
| Informally consider reliability of coding throughout the piloting process | Informal methods are an alternative to quantifying agreement. Reviewers can, for example, document problems while coding and discussing these at a review group meeting. |

**Dimension: Applying data extraction forms**

| Item | Rationale for inclusion in our analysis |
| --- | --- |
| Data extraction should be conducted by at least two people | Empirical evidence shows that extraction errors are common [4]. Data extraction by two people reduces the risk of errors [5]. |
| Data extraction should be conducted by at least two people independently (parallel extraction) | Data can be extracted by two reviewers independently, which is the ideal method to reduce extraction errors, albeit very labour intensive. |
| Data can be extracted by one individual while the second individual checks for accuracy and completeness (double-checking) | Data can be extracted by one reviewer and checked by a second reviewer for accuracy. This method is more prone to error than independent parallel extraction [5]. The advantage is a reduced workload. |
| Use parallel extraction for critical items (e.g. Risk of Bias, Outcome-Daten) and double-checking for non-critical items | This method provides a compromise between independent parallel extraction and double-checking, although it may be debatable which data are critical and to the best of our knowledge there is little empirical evidence on the frequency of extraction errors for non-outcome data [4]. |
| Validate a random sample of data by a third investigator for additional quality assurance | Sometimes it is recommended that a third reviewer validates a sample of extraction forms. This may be particularly useful, when the main data extractors are less experience or in large reviews with many extractors. Here the main investigator could look at extractions from different pairs of extractors for example, to get an idea of the consistency. |
| Data extraction by reviewers with complementary expertise (e.g. clinical and methodological) | As with development and piloting of extraction forms there may be benefits of having reviewers with complementary expertise extract data, typically someone with content expertise and someone with methodological expertise, for example. |
| Data extraction by at least one reviewer with expertise in systematic review methods/data extraction |
| Quantify agreement using a reliability measure such as Cohen's kappa | Some sources mention the possibility of or recommend measuring agreement of the data extracted from the included studies. One of the merits may be that readers of the final review get a somewhat objective idea of the reliability of the extracted data. |
| Quantify agreement using a reliability measure (e.g. Cohen's kappa) only for critical items such as risk of bias items or outcome data | Given the additional work required in quantifying agreement and the unclear value, it is sometimes recommended that if this is done, it should be restricted to important extraction items. |
| Informally consider reliability of coding in the data extraction phase | Instead of quantifying agreement formally, reviewers can use informal methods such as documenting problems while coding and discussing these at review group meetings, for example. |
| Develop explicit procedures or rules for resolving disagreements | When two reviewers extract data and discordances arise it is customary to have methods in place that ensure disagreements are resolved appropriately. This is often done by discussion or arbitration with a third reviewer, although other methods are possible. |
| Report who was involved in data extraction | While this item refers to reporting, we have included it as it may provide additional information on some of the above-mentioned aspects, when these are not specifically reported. |
| Document disagreements and how they were resolved | Keeping a paper trail of the disagreements and how they were resolved, help if one must review the data at a later stage or post publication, for example. |

**References**

1. von Elm E, Poglia G, Walder B, Tramèr MR. Different patterns of duplicate publication: an analysis of articles used in systematic reviews. JAMA. 2004;291:974-980.

2. Tramèr MR, Reynolds DJ, Moore RA, McQuay HJ. Impact of covert duplicate publication on meta-analysis: a case study. BMJ. 1997;315:635-640.

3. Kirkham JJ, Dwan KM, Altman DG, et al. The impact of outcome reporting bias in randomised controlled trials on a cohort of systematic reviews. BMJ. 2010;340:c365.

4. Mathes T, Klaßen P, Pieper D. Frequency of data extraction errors and methods to increase data extraction quality: a methodological review. BMC Med Res Methodol. 2017;17:152.

5. Robson RC, Pham B, Hwee J, et al. Few studies exist examining methods for selecting studies, abstracting data, and appraising quality in a systematic review. J Clin Epidemiol. 2019;106:121-135.
