# Supplementary material for: Development, testing and use of data extraction forms in systematic reviews: a review of methodological guidance
Source: BMC Med Res Methodol. 2020 Oct 19;20:259. doi: 10.1186/s12874-020-01143-3 (PMC7574308; doi:10.1186/s12874-020-01143-3)
Supplement: Supplementary file 4 — Additional file 4. List of included documents [file 12874_2020_1143_MOESM4_ESM.docx]

**Additional file 4: lists of included documents reviewed for recommendations on development, piloting and application of data extraction sheets**

**Handbooks from systematic review organisations**

Aromataris E, Munn Z (Editors)*.* Joanna Briggs Institute Reviewer's Manual. The Joanna Briggs Institute; 2017. <https://reviewersmanual.joannabriggs.org/> Accessed 04 June 2020.

Centre for Reviews and Dissemination. CRD’s guidance for undertaking reviews in health care. York: York Publishing Services Ltd. 2009.

Higgins JPT, Thomas J, Chandler J, Cumpston M, Li T, Page MJ, et al., editors. Cochrane Handbook for Systematic Reviews of Interventions version 6.0. Cochrane; 2019. [www.training.cochrane.org/handbook](http://www.training.cochrane.org/handbook) Accessed 04 June 2020.

Institute of Medicine. Finding What Works in Health Care: Standards for Systematic Reviews. Washington, DC: The National Academies Press; 2011.

**HTA agency manuals, handbooks, guidance documents and standard operating procedures**

Fröschl B, Bornschein B, Brunner-Ziegler S, Conrads-Frank A, Eisenmann A, Gartlehner G, et al. Methodenhandbuch für Health Technology Assessment. Gesundheit Österreich GmbH; 2012. <https://jasmin.goeg.at/121/> Accessed 19 Feb 2019.

Gartlehner G. (Internes) Manual Abläufe und Methoden. Ludwig Boltzmann Institut für Health Technology Assessment (LBI-HTA); 2007. <http://eprints.aihta.at/713/> Accessed 19 Feb 2019.

Health Information and Quality Authority (HIQA). Guidelines for the retrieval and interpretation of economic evaluations of health technologies in Ireland. HIQA; 2014. [https://www.hiqa.ie/reports-and-publications/health-technology-assessments/guidelines-interpretation-economic Accessed 19 Feb 2019](https://www.hiqa.ie/reports-and-publications/health-technology-assessments/guidelines-interpretation-economic%20Accessed%2019%20Feb%202019).

Institute for Clinical and Economic Review (ICER). A Guide to ICER’s Methods for Health Technology Assessment. ICER; 2018. <https://icer-review.org/methodology/icers-methods/icer-hta-guide_082018/> Accessed 19 Feb 2019.

International Network of Agencies for Health Technology Assessment (INAHTA). A checklist for health technology assessment reports. INAHTA; 2007. <http://www.inahta.org/hta-tools-resources/briefs/> Accessed 19 Feb 2019.

Malaysian Health Technology Assessment Section (MaHTAS). Manual on health technology assessment. 2015. <https://www.moh.gov.my/moh/resources/HTA_MANUAL_MAHTAS.pdf?mid=636>

**Textbooks**

Bettany-Saltikov, J. How to do a systematic literature review in nursing: a step-by-step guide. Berkshire: McGraw-Hill Education; 2012.

Booth A, Papaioannou D, Sutton A. Systematic Approaches to a Successful Literature Review. London: Sage Publications Ltd; 2012.

Cooper HM. Synthesizing Research: A Guide for Literature Reviews. Thousand Oaks: Sage Publications Inc; 1998.

Egger M, Smith GD, Altman DG. Systematic Reviews in Health Care: Meta-Analysis in Context. 2nd ed. London: BMJ Publishing Group; 2001.

Foster MJ, Jewell ST. Assembling the pieces of a systematic review: a guide for librarians. Lanham: Rowman & Littlefield; 2017.

Holly C, Salmond SW, Saimbert M. Comprehensive systematic review for advanced nursing practice. New York: Springer Publishing Company; 2012.

Khan KS, Kunz R, Kleijnen J, Antes G. Systematic Reviews to Support Evidence-based Medicine. London: Royal Society of Medicine Press; 2003.

Mulrow CD, Cook D. Systematic reviews: synthesis of best evidence for health care decisions. Philadelphia: ACP Press; 1998.

Petticrew M, Roberts H. Systematic reviews in the social sciences: a practical guide. Malden: Blackwell Publishing; 2006.

Pope C, Mays N, Popay J. Synthesizing Qualitative and Quantitative Health Evidence. Maidenhead: McGraw Hill; 2007.

Sharma R, Gordon M, Dharamsi S, Gibbs T. Systematic reviews in medical education: A practical approach: AMEE Guide 94. Dundee: Association for Medical Education in Europe; 2015.

**Journal articles identified from searches of the Scientific Resource Center’s Methods Library, Cochrane Methodology Register and Medline**

Furlan AD, Malmivaara A, Chou R, et al. 2015 Updated Method Guideline for Systematic Reviews in the Cochrane Back and Neck Group. Spine. 2015;40:1660-73.

Li T, Vedula SS, Hadar N, Parkin C, Lau J, Dickersin K. Innovations in data collection, management, and archiving for systematic reviews. Ann Intern Med. 2015;162:287-94.

Munn Z, Tufanaru C, Aromataris E. JBI’s systematic reviews: data extraction and synthesis. Am J Nurs. 2014;114:49-54.

Pullin AS, Stewart GB. Guidelines for systematic review in conservation and environmental management. Conserv Biol. 2006;20:1647-1656.

Stock WA, Goméz Benito J, Balluerka Lasa N. Research synthesis. Coding and conjectures. Eval Health Prof. 1996;19:104-17.
