# Supplementary material for: Development, testing and use of data extraction forms in systematic reviews: a review of methodological guidance
Source: BMC Med Res Methodol. 2020 Oct 19;20:259. doi: 10.1186/s12874-020-01143-3 (PMC7574308; doi:10.1186/s12874-020-01143-3)
Supplement: Supplementary file 5 — Additional file 5. Recommendations for non-interventional reviews [file 12874_2020_1143_MOESM5_ESM.docx]

**Additional file 5: recommendations on non-interventional review types**

**Table 1: Development of data collection forms**

| **Source** | **Item** | | | | | | | | | | |
| --- | --- | --- | --- | --- | --- | --- | --- | --- | --- | --- | --- |
|  | **Plan in advance which data are needed** | **Develop a customized or adapted form** | **Use generic form** | **Ensure consistent and clear coding and response options** | **Provide detailed instructions** | **Number of reviewers involved in developing the form** | **Involve reviewers with complementary expertise** | **Involve reviewers with experience in systematic review methods** | **Link multiple reports of the same study** | **Develop mechanism for recording, assessing and correcting data entry errors** | **Develop a strategy for obtaining data** |
| **JBI** | | | | | | | | | | | |
| SRs of qualitative evidence | + | / | / | / | / | / | / | / | / | / | / |
| SRs of text and opinion | + | o | + | / | + | / | / | / | / | / | / |
| SRs of prevalence and incidence | + | + | / | / | / | / | / | / | / | / | / |
| SRs of economic evidence | / | o | + | / | / | / | / | / | / | / | / |
| SRs of etiology and risk | + | / | / | / | / | / | / | / | / | / | / |
| Mixed methods SRs | / | o | o | / | / | / | / | / | / | / | + |
| Diagnostic test accuracy SRs | + | / | / | / | / | / | / | / | / | / | / |
| Umbrella reviews | + | o | + | / | / | / | / | / | / | / | gs |
| Scoping reviews | + | o | o | / | / | / | / | / | / | / | / |
| **HTA agencies** | | | | | | | | | | | |
| HIQA Guidelines for the Retrieval and Interpretation of Economic Evaluations | / | + | / | / | / | / | / | / | / | / | / |

Legend: + = recommended; o = optional; gs = general statement; / = not mentioned; SRs = systematic reviews; JBI: Joanna Briggs Institute; HTA: Health Technology Assessment

**Table 2: Piloting of data collection forms**

| **Source** | **Item** | | | | | | | | | |
| --- | --- | --- | --- | --- | --- | --- | --- | --- | --- | --- |
|  | **Train data extractors in using the form** | **Pilot test form using a sample of studies** | **(Partially) repeat piloting if major changes are made during the review** | **In case of modifications to the data collection form, re-check reports that have already undergone data extraction** | **Involve reviewers with complementary expertise** | **Involve reviewer with experience in systematic review methods** | **Quantify agreement using a reliability measure such as Cohen's kappa** | **If agreement is quantified using a reliability, do this only for critical items** | **Repeat the piloting process until a specified agreement is reached** | **Informally consider reliability of coding while piloting** |
| **JBI** | | | | | | | | | | |
| SRs of qualitative evidence | / | / | / | / | / | / | / | / | / | / |
| SRs of text and opinion | / | / | / | / | / | / | / | / | / | / |
| SRs of prevalence and incidence | / | / | / | / | / | / | / | / | / | / |
| SRs of economic evidence | / | / | / | / | / | / | / | / | / | / |
| SRs of etiology and risk | / | / | / | / | / | / | / | / | / | / |
| Mixed methods SRs | / | / | / | / | / | / | / | / | / | / |
| Diagnostic test accuracy SRs | / | + | / | / | / | / | / | / | / | / |
| Umbrella reviews | / | + | / | / | / | / | / | / | / | / |
| Scoping reviews | / | + | / | / | / | / | / | / | / | / |
| **HTA agencies** | | | | | | | | | | |
| HIQA Guidelines for the Retrieval and Interpretation of Economic Evaluations | / | / | / | / | / | / | / | / | / | / |

Legend: + = recommended; / = not mentioned; SRs = systematic reviews; JBI: Joanna Briggs Institute; HTA: Health Technology Assessment

**Table 3: Data extraction**

| **Source** | **Item** | | | | | | | | | | | | | |
| --- | --- | --- | --- | --- | --- | --- | --- | --- | --- | --- | --- | --- | --- | --- |
|  | **Data extraction should be conducted by at least two people** | **Data extraction by at least two people independently (parallel extraction)** | **Data extracted by one individual accuracy checks by a second (double-checking)** | **Parallel extraction for critical items and double-checking for non-critical items** | **Validation of a random sample of data by a third investigator** | | **Data extraction by reviewers with complementary expertise** | **Data extraction by at least one reviewer with expertise in systematic review methods** | **Quantify agreement using a reliability measure such as Cohen's kappa** | **If agreement is quantified, do this only for critical items** | **Informally consider reliability of coding throughout the review process** | **Explicit procedures or rules for resolving disagreements** | **Report who was involved in data extraction** | **Document disagreements and how they were resolved** |
| **JBI** |  |  |  |  |  |  | |  |  |  |  |  |  |  |
| SRs of qualitative evidence | + | + | / | / | / | / | | / | / | / | / | + | + | / |
| SRs of text and opinion | + | + | / | / | / | / | | / | / | / | / | + | + | / |
| SRs of prevalence and incidence | + | + | / | / | / | / | | / | / | / | / | + | + | / |
| SRs of economic evidence | o | / | / | / | / | / | | / | / | / | / | o | / | / |
| SRs of etiology and risk | / | / | / | / | / | / | | / | / | / | / | / | / | / |
| Mixed methods SRs | + | / | / | / | / | / | | / | + | / | / | + | / | / |
| Diagnostic test accuracy SRs | + | + | / | / | / | / | | / | / | / | / | + | + | / |
| Umbrella reviews | + | + | / | / | / | / | | / | / | / | / | + | + | / |
| Scoping reviews | / | / | / | / | / | / | | / | / | / | / | / | / | / |
| **HTA agencies** |  |  |  |  |  |  | |  |  |  |  |  |  |  |
| HIQA Guidelines for the Retrieval and Interpretation of Economic Evaluations | + | + | / | / | / | / | | / | + | / | + | + | + | / |

Legend: + = recommended; / = not mentioned; SRs = systematic reviews; JBI: Joanna Briggs Institute; HTA: Health Technology Assessment
